# Impact of Glucocorticoid Receptor Density on Ligand-Independent Dimerization, Cooperative Ligand-Binding and Basal Priming of Transactivation: A Cell Culture Model

**DOI:** 10.1371/journal.pone.0064831

**Published:** 2013-05-22

**Authors:** Steven Robertson, Johann M. Rohwer, Janet P. Hapgood, Ann Louw

**Affiliations:** 1 Department of Biochemistry, University of Stellenbosch, Matieland, Stellenbosch, Republic of South Africa; 2 Department of Molecular and Cell Biology, University of Cape Town, Cape Town, Republic of South Africa; Kaohsiung Chang Gung Memorial Hospital, Taiwan

## Abstract

Glucocorticoid receptor (GR) levels vary between tissues and individuals and are altered by physiological and pharmacological effectors. However, the effects and implications of differences in GR concentration have not been fully elucidated. Using three statistically different GR concentrations in transiently transfected COS-1 cells, we demonstrate, using co-immunoprecipitation (CoIP) and fluorescent resonance energy transfer (FRET), that high levels of wild type GR (wtGR), but not of dimerization deficient GR (GRdim), display ligand-independent dimerization. Whole-cell saturation ligand-binding experiments furthermore establish that positive cooperative ligand-binding, with a concomitant increased ligand-binding affinity, is facilitated by ligand-independent dimerization at high concentrations of wtGR, but not GRdim. The down-stream consequences of ligand-independent dimerization at high concentrations of wtGR, but not GRdim, are shown to include basal priming of the system as witnessed by ligand-independent transactivation of both a GRE-containing promoter-reporter and the endogenous glucocorticoid (GC)-responsive gene, GILZ, as well as ligand-independent loading of GR onto the GILZ promoter. Pursuant to the basal priming of the system, addition of ligand results in a significantly greater modulation of transactivation potency than would be expected solely from the increase in ligand-binding affinity. Thus ligand-independent dimerization of the GR at high concentrations primes the system, through ligand-independent DNA loading and transactivation, which together with positive cooperative ligand-binding increases the potency of GR agonists and shifts the bio-character of partial GR agonists. Clearly GR-levels are a major factor in determining the sensitivity to GCs and a critical factor regulating transcriptional programs.

## Introduction

The glucocorticoid receptor (GR) is a ligand-dependent transcription factor that mediates the effects of both endogenous glucocorticoids (GCs) as well as synthetic GCs used to treat inflammatory diseases [1]–[3]. Despite the fact that the GC concentration in the bloodstream is equal at any specific time point and that GR is present in every major tissue [4], [5], there are considerable tissue specific [6], inter-individual [7], [8] and diseased compared to healthy tissue [9] differences in GC response. GR levels [10] are amongst the factors [11]–[19] shown to influence the degree and tissue specificity of transcription via the GR.

GR is down-regulated in response to GC exposure in most tissues [20]–[23] and in some tissues down-regulation is linked to aging [24], [25], exercise [24] and psychological stress [26], [27], while up-regulation in some tissues is linked to HIV infection [28], muscle sepsis [9], dietary restriction [29], adrenalectomy [30] and cancer [10]. In addition, GR expression levels differ greatly between tissues [5], [31] and inter-individual variances are found within the same tissue type [8], [32]. Physiologically, decreased GR levels are associated with GC resistance in rheumatoid arthritis [33], [34], lupus nephritis [35], bronchial asthma [36] and sepsis [37], while increased GR levels are linked to GC sensitivity in a range of cancers.

In most cases increased GR levels have simply been linked to an increase in the efficacy of ligand, however, raising GR concentration has also been shown to result in enhanced potency of GR-mediated transcription [38]–[40] and bio-character shifts of partial agonists to more efficacious ligands [41], [42]. In addition, a shift from non-cooperative to positive cooperative ligand-binding as GR levels increase has also been shown *in vitro* [43], which would suggest an increase in affinity. However, the relationship between GR levels and ligand-binding affinity is controversial with some authors indicating that increased GR levels result in increased affinity [31], while others find a decrease in affinity [44], [45].

Although studies on the influence of GR concentration on transcription have shown clear shifts in the potency of agonists and bio-character of partial agonists, none have defined the specific GR concentrations at which these shifts occur nor have they attempted to correlate the changes in GR-induced transcription with a change in ligand-binding affinity or shift from non-cooperative ligand-binding to positive cooperative ligand-binding. Furthermore, the molecular mechanism responsible for cooperative ligand-binding has not been demonstrated experimentally. Here we show that ligand-independent dimerization of the GR at high concentrations is responsible for the phenomena of positive cooperative ligand-binding and concomitant increase in affinity. Furthermore, we demonstrate that ligand-independent dimerization significantly increases the potency of GC-induced transactivation, as well as causing a shift in the bio-character of partial agonists. However, our work reveals that the magnitude of the increase in transactivation potency is greater than would be expected from the observed increase in ligand-binding affinity alone and that basal priming of the GR-signaling system through ligand-independent loading of the GR onto DNA with consequent ligand-independent transactivation contributes to the increase in transactivation potency.

## Materials and Methods

### Reagents

Dexamethasone (DEX), cortisol (F), progesterone (Prog), medroxyprogesterone acetate (MPA) and mifepristone (RU486) were purchased from Sigma. Compound A (CpdA) or 2-(4-acetoxyphenyl)-2-chloro-N-methylethylammonium chloride was synthesized as described previously [46]. The [^3^H]-DEX (specific activity of 68–85 Ci/mmol) was obtained from AEC Amersham Biosciences.

### Plasmids

pGL2-basic (empty vector) was purchased from Promega. pRS-hGRα (GRwt) was a gift from R. M. Evans [47], pHisGRA458T (GRdim) and pEFFlaghGRα (Flag-GR, molecular mass, 96 kDa) [48] were gifts from K. De Bosscher (University of Ghent, Belgium). pEGFP-C2-GR (GFP-GR, molecular mass, 128.5 kDa) was provided by S. Okret (Karolinska Institute, Sweden) [49]. ECFP-hGRα (CFP-GR) and pEYFP-hGRα (YFP-GR) were gifts from J. Cidlowski [50]. pTAT-GRE2-E1b-luc was a gift from G. Jenster [51] and pΔODLO was a gift from D. Pearce [52]. pEGFP-C2-GRA477T (GFP-GRdim) was cloned by excising the wild type GR from pEGFP-C2-GR with *Xma*I and *Sal*I and replacing it with the mutated GRdim sequence from pHisGRA458T. The presence of the mutation was confirmed through sequencing (primer used: 5′-AGCTTCAGGATGTCATTATGGAG-3′).

### Cell culture and DEAE-dextran transfection

COS-1 cells (purchased from ATCC) were cultured in DMEM with 2 mM glutamine, 44 mM sodium bicarbonate, and 1 mM sodium pyruvate (**un-supplemented DMEM**) supplemented with 10% fetal calf serum (FCS), 100 IU/ml penicillin and 100 µg/ml streptomycin (**complete DMEM**). Cells were transfected with the indicated amounts of GR using the DEAE-dextran method [53] in 10 cm plates (2×10^6^ cells/10-cm plate) made up to 12 µg total plasmid with empty vector unless otherwise stated. GR levels were monitored throughout using whole-cell saturation binding, immunoblotting and fluorescent intensity (Fig. S1 in File S1). Pixels from digitized immunoblots were used to compare the expression levels of transiently transfected GR to levels determined in saturation binding assays (cpm/mg protein) and revealed a good correlation (R^2^ = 0.97) between the two methods of determining GR concentration (Fig.S1A in File S1) in accordance with a comparative study by O′Donnell [30], which compared immunoblots and radioactive ligand-binding as means of GR quantification. For FRET assays, using fluorescently-tagged GRs, CFP fluorescent signal was used to quantify GR levels (Fig. S1B in File S1).

### Whole-cell Saturation binding

Cells were transfected with 40 ng (low), 400 ng (medium) or 12 µg (high) GRwt, or 40 ng (low) or 12 µg (medium) GRdim. Cells were replated 24 h later (1x10^5^ cells/well in 24-well plates) in medium with 10% dextran-coated charcoal stripped FCS (Highveld Biologicals, South Africa) and 1% Pen/Strep (**charcoal-stripped DMEM**). Twenty four hours after replating cells were incubated for 4 h at 37°C with increasing concentrations of [^3^H]-DEX only (total binding), or [^3^H]-DEX together with a constant concentration of 60 µM unlabeled DEX (non-specific binding) in un-supplemented DMEM. Washing and lysis of cells was as described in Robertson *et al* [48]. Binding was normalized to protein concentration [54]. Specific binding (total binding − nonspecific binding) was plotted against nM [3H]-DEX and curves fitted using one site binding hyperbola to obtain Kd and Bmax values. Bmax values and a counting efficiency of 43% was used to calculate fmol GR/mg protein. To obtain Hill slopes specific binding was plotted against logM [3H]-DEX and curves fitted using sigmoidal dose-response (variable slope).

### Immunoblotting

COS-1 cells were transfected with low, medium or high GRwt or low or medium GRdim for promoter-reporter, real-time PCR and ChIP studies. Twenty-four hours after transfection cells were replated either for immunoblotting or for promoter-reporter, real-time PCR or ChIP studies. For immunoblotting cells were replated into 10 cm tissue culture plates (2.5×10^6^ cells/plate) in charcoal-stripped DMEM. Twenty-four hours after replating and without induction cells were washed twice with PBS before being lysed on ice in BufferA (10 mM Hepes pH 7.5 (Invitrogen), 1.5 mM MgCl2, 10 mM KCl, 0.1% Nonidet P-40 (Roche Applied Science), and Complete Mini protease inhibitor mixture (Roche Applied Science)). After two cycles of freeze-thaw the lysate was centrifuged at 14,000× g for 15 min, and the supernatant collected. In addition, Co-IP input lysates were also immunoblotted to control for GR levels. Protein concentrations of all lysates were determined using the Bradford method [54]. Protein (20 µg) was loaded and separated on a 10% SDS-PAGE gel. Following electrophoresis, proteins were electroblotted and transferred to Hybond-ECL nitrocellulose membrane (Amersham Biosciences), which were probed for GR with H-300 anti-GR (Santa Cruz Biotechnology) diluted 1∶3000 in 5% (w/v) casein in TBST buffer followed by ECL peroxidase-labelled anti-rabbit antibody (AEC-Amersham Biosciences) diluted 1∶10000 in 5% (w/v) casein in TBST buffer. Blots were visualized with ECL Western blotting detection reagents (GE Healthcare) on Hyperfilm (Amersham Biosciences). Densitometric analysis of the immunoblots was carried out using UN-SCAN-IT gel 6.1 software (Silk Scientific).

### Promoter-reporter transactivation

Cells were transfected with low, medium or high GRwt, low or medium GRdim and 3000 ng pTAT-GRE2-E1b-luc filled to 14550 ng total plasmid with empty vector. Cells were replated 24 h later into 96-well plates (4×10^4^ cells/well) in charcoal-stripped DMEM. Twenty-four hours after replating, cells were induced with vehicle (ethanol) or increasing concentrations (10^−14^ to 10^−5^ M) of DEX, cortisol, MPA, or RU486 in charcoal-stripped DMEM for 24 h. Cells were lysed with 30 µl of passive lysis buffer (Promega), and subjected to a freeze thaw cycle. Luciferase activity was determined using a luciferase assay kit (Promega). Light emission was measured in a Veritas microplate luminometer (Turner Biosystems). Luciferase relative light units were normalized against protein concentrations [54]. Sigmoidal dose-response curves were fit to the experimental data which generated basal induction (bottom), maximal induction (top-bottom), fold-induction (top/bottom), and log EC_50_.

### Co-immunoprecipitation (Co-IP)

Cells were transfected with low levels of GR (34.22 ng Flag-GR and 4.28 ng GFP-GRdim or GFP-GRwt); medium levels of GR (342.2 ng Flag-GR and 42.8 ng GFP-GRdim or GFP-GRwt) or high levels of GR (10266 ng Flag-GR and 1284 ng GFP-GRdim or GFP-GRwt). Twenty-four hours later cells were steroid starved in charcoal-charcoal stripped DMEM for 24 h then treated with vehicle (ethanol), 10^−6^ M DEX, or 10^−5^ M CpdA for 1 h. Cells were lysed and protein determination was carried out as described in Robertson *et al.* [48]. Cell lysate (600 µg of protein from the low GR samples or 200 µg of protein from medium and high GR samples) was added to 30 µl EZview Red ANTI-FLAG M2 Affinity Gel beads (Sigma), pre-washed 4 times with Buffer A in the presence of 2.5% (w/v) casein protein and Complete Mini protease inhibitor mixture (Roche). The final volume was topped up to 400 µl/sample with Buffer A containing 2.5% (w/v) casein protein and Complete Mini protease inhibitor mixture and rotated for 16 h at 4°C. Beads were washed and immune precipitates prepared and processed as described in Robertson *et al.* [48]. Densitometric analysis of the immunoblots was carried out using UN-SCAN-IT gel 6.1 software (Silk Scientific) and GFP-GR pull down was normalized against their respective Flag-GR levels.

### Fluorescence resonance energy transfer (FRET)

Cells were transfected with low levels of GR (19.25 ng CFP-GR and 19.25 ng YFP-GR), medium levels of GR (192.5 ng CFP-GR and 192.5 ng YFP-GR) or high levels of GR (5775 ng CFP-GR and 5775 ng YFP-GR). Twenty-four hours after transfection cells were replated (3×10^4^ cells/well) into 8-well Lab-Tek chambered coverglass plates (Nunc, Denmark) in charcoal-stripped DMEM. Twenty-four hours after replating cells were analyzed in the temperature-controlled chamber (37°C) of an IX-81 Olympus Cells system with YFP, CFP and FRET filter sets as described in Robertson *et al.* [48]. Cells were selected which expressed both CFP-GR and YFP-GR. Cells were induced with 10^−6^ M DEX in un-supplemented DMEM. CFP, YFP and FRET images were taken every minute over a 30 min period. An exposure time of 1500 ms at 100% light intensity was used and the entire cell area as defined by the cellular membrane was selected as the region of interest. The F-don signal (CPF) was used to select cells for analysis (Fig. S1B in File S1). Cells with an F-don emission of 0–600 were selected from the low GR population, F-don signals between 600–1200 from the medium GR population and F-don of >1200 from the high GR population. The FRET signal was corrected for bleed through as described in Robertson *et al.* [48].

### Real-time PCR

Cells were transfected with low, medium or high GRwt or low or medium GRdim and replated 24 h after transfection (5×10^5^/well/12 well plate) in charcoal-stripped DMEM. Twenty-four hours after replating cells were induced with vehicle (ethanol) or a range of DEX concentrations for 8 h. RNA isolation, cDNA synthesis and quantitative PCR was carried out on the glucocorticoid induced leucine zipper (GILZ) and glyceraldehyde 3-phosphate dehydrogenase (GAPDH) as housekeeping gene as previously described [55]. The GILZ primers were purchased through QuantiTect primers (Qiagen) and have an amplicon size of 69 bp. The GAPDH primers were synthesized by Integrated DNA Technologies (forward, 5′- TGA ACG GGA AGC TCA CTG G-3′ and reverse 5′-ATT CGT TGT CAT ACC AGG-3′) (13) and have an amplicon size of 307 bp.

### Chromatin Immunoprecipitation (ChIP) Assay

COS-1 cells were transiently transfected in T75 flasks with no GR, low, medium or high GRwt and low or medium GRdim and filled to 12 µg total plasmid with empty vector. Cells were replated 24 h later onto 10 cm plates (3.5×10^6^ cells/well) in charcoal-stripped DMEM. Twenty-four hours after replating cells were induced with vehicle (ethanol) or 10^−6^ M DEX for 1 h, before being cross-linked with 1% formaldehyde. The formaldehyde was quenched with glycine, where after the cells were washed and re-suspended in nuclear lysis buffer. Cells were sonicated on 75% power, for 20 cycles at 20 sec per cycle, with 20 sec intervals between pulses, using the Misonix Sonicator® S-4000 sonicator with cup horn. After sonication, the lysates were centrifuged to remove the cellular debris. An aliquot of the supernatants (15 µg of chromatin) was removed and used as input, while 50 µg of the chromatin was immunoprecipitated overnight with 5 µg of anti-GR antibody (H300, Santa Cruz Biotechnology), as well as an anti-IgG antibody (Santa Cruz Biotechnology) as a negative control. After incubation with protein A/G beads (Santa Cruz Biotechnology) and extensive washing, the immunoprecipitated DNA was eluted from the beads using elution buffer. After the cross-links were reversed overnight, the samples were treated with proteinase K (Roche) and the DNA was purified using the Qiagen PCR purification kit. The purified DNA was subjected to quantitative real-time PCR, using specific primers for the promoter of the endogenous GILZ gene, spanning the equivalent of GREs 3–6 (GILZ F, 5′-AGT TAA GCT CCT GAT TTA AGA AG-3′; GILZR, 5′-CCC GAT CTC AGG ACA TTC-3′) and based on homology among the human, chimp, and rhesus monkey GILZ promoter sequences [56].

### Statistical analysis

Statistical analyses were carried out using GraphPad Prism software, using one-way analysis of variance (ANOVA) with either Bonferroni, Dunnett or Newman-Keuls post-tests or two tailed unpaired t tests as indicated in legends.

## Results

### Increasing GR concentration results in a greater than expected increase in efficacy and potency of promoter-reporter assay

**Figure 1:**
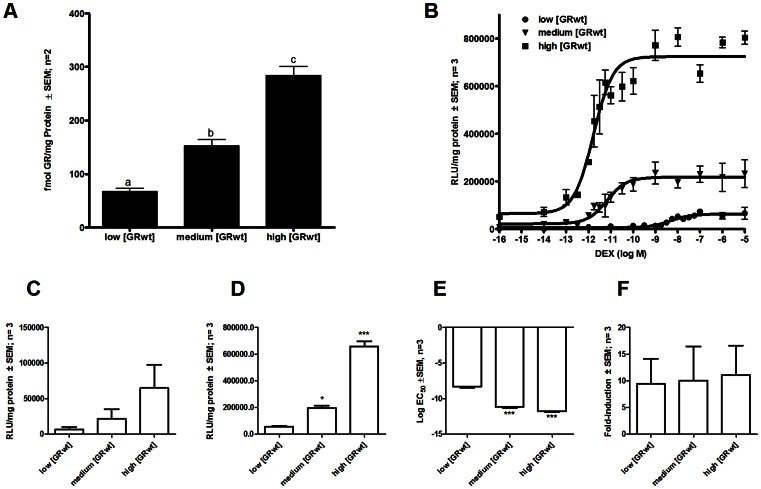
Linear increase in GRwt concentration leads to exponential increase in potency of transactivation. (***A***) COS-1 cells were transiently transfected with GRwt (low, medium or high levels) to establish three statistically different, yet physiologically relevant, GRwt concentrations. Saturation binding was used to determine specific binding from which fmol GR/mg protein was calculated. Statistical analysis was carried out using one-way ANOVA followed by Newman-Keuls multiple comparisons post-test. Conditions with different letters are statistically different from one another (P<0.05). Results represent two independent experiments performed in triplicate (±SEM). (***B***) COS-1 cells were transiently transfected with GRwt (low, medium or high levels) and the GRE-containing promoter-reporter pTAT-GRE2-E1b-luc. Cells were induced with ethanol or increasing concentrations (10^−14^ to 10^−5^ M) of DEX for 24 hours. Luciferase relative light units (RLU) were normalized against protein concentrations and sigmoidal dose-response curves fit to the experimental data to generate (***C***) basal (bottom) and (***D***) maximal induction (top-bottom) as well as (***E***) log EC_50_ and (***F***) fold-induction (top/bottom). Statistical analysis for ***C*** **–** ***F*** was carried out using one-way ANOVA followed by Dunnett's post-test against the low GRwt condition. Results represent a minimum of three independent experiments performed in triplicate (±SEM).

Three physiologically relevant, yet statistically (P<0.05) different, concentrations of human GR wild type (GRwt), were established by transient transfection in COS-1 cells. These GR concentrations were designated as low, medium and high GRwt concentrations and saturation binding (Fig. 1A) established (i) that these GR concentrations of 67.0±8.8, 152.6±16.8, and 283.9±23.8 fmol GR/mg protein, respectively, fall within the physiological range of GR concentrations of 4–900 fmol GR/mg protein [28], 57, and (ii) that the concentrations increase two-fold from low to medium GRwt concentration, and four-fold from low to high GRwt concentration.

However, fluorescence microscopy showed that roughly 20% of cells in a population were transfected in our studies (results not shown). Thus in order to acknowledge this fact we have calculated the average of the fmol GR/mg protein in transfected cells to be five-fold higher, i.e. 335, 763 and 1420 fmol/mg protein for low, medium and high GR levels, respectively, while the GR/cell is 26200, 59600 and 111000, respectively. GR reported in bone marrow ranges from 1106 to 27000 GR/cell [8] while another study revealed GR levels as high as 893 fmol GR/mg protein in healthy skin, rising to 2777 fmol GR/mg protein in the skin of AIDS patients [28]. Cytotrophoblasts (epithelial stem cells) have been shown to contain GR concentrations as high as 16200 fmol GR/mg protein [58] well above the highest concentration achieved in our system. Furthermore, MCF-7, a breast cancer cell line, has been reported to contain 29995 GR/cell [59], while SiHa, a uterine cervical cancer cell line, and Hep3B, a hepatoma cell line, contain 81000 and 43000 GR/cell, respectively [10]. We can therefore argue that our low GR concentrations reflect physiological GR levels when compared to GR levels in bone marrow [8] or MCF-7 cells [59], while our medium and high GR levels reflect physiological GR levels in normal and AIDS patient skin [28] or Hep3B and SiHa cells [10], respectively.

To assess the effect of GR concentration on transcription, DEX transactivation of a multiple glucocorticoid-response element (GRE) containing promoter-reporter, pTAT-GRE2-E1b-luc, was studied at the three GRwt concentrations established (Fig. 1B). This type of promoter represents the majority of direct GR DNA interactions [60] and provides a robust transactivation response. The promoter of this construct consists of two copies of the GRE from the tyrosine amino transferase gene (TAT) as well as the TATA box from the E1b promoter, which serves as a generic docking site for secondary transcription factors [51], [61]. Data from the dose response curves indicate larger than expected increases in basal induction (Fig. 1C) and efficacy (Fig. 1D), as well as in potency (Fig. 1E), but not in fold-induction (Fig. 1F), due to increased GRwt concentrations. Specifically, basal induction increased three- and ten-fold, efficacy four- and 12-fold, and potency (EC_50_) 650- and 2600-fold, respectively, as GRwt concentration increased only two- and four-fold. In contrast, fold-induction remained relatively constant at between 9-and 11-fold for all GRwt concentrations. The fact that the magnitude of the increases in dose-response parameters were greater than predicted from the increase in GRwt concentrations alone, prompted us to further investigate the mechanism whereby increased GRwt concentrations could affect GR signalling. Especially the exponential increase in potency of transactivation at higher GRwt concentrations suggested a co-operative mechanism, which may require more than one ligand-binding site, and we thus hypothesised that increased GRwt concentrations may lead to ligand-independent dimerization of the GRwt and cooperative ligand-binding.

### The ability of the GR to dimerize is a prerequisite for positive cooperative ligand-binding

A previous study [43] had shown *in vitro* that positive cooperative ligand-binding occurs at higher concentrations of rat GRwt. We sought to confirm this finding with human GRwt. Furthermore, as cooperative ligand-binding presupposes the presence of more than one ligand-binding site, where ligand-binding to the first site facilitates a conformation change that results in the cooperative binding of the second ligand [62], we wanted to establish that dimerization of the GR is a prerequisite for cooperative ligand-binding. To this end we included the DNA binding domain (DBD) dimerization-loop mutant GR (GRdim) [63] in our study.

**Figure 2:**
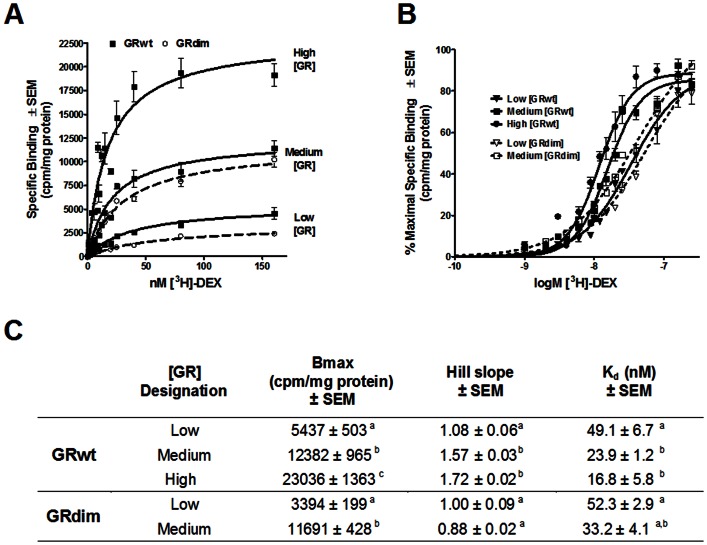
Increased concentration of GRwt, but not GRdim, displays cooperative ligand-binding. COS-1 cells were transiently transfected with GRwt (low, medium or high) or GRdim (low or medium) before saturation binding was carried out with the depicted [^3^H]-DEX concentrations. *(* ***A*** *)* Specific binding was plotted against nM [^3^H]-DEX and curves fitted using one site binding hyperbola to obtain K_d_ and Bmax values. (***B***) Specific binding was plotted against logM [^3^H]-DEX and curves fitted using sigmoidal dose-response (variable slope) to obtain Hill slopes. (***C***) Summary table of saturation binding results. Statistical analysis of maximal binding (Bmax), Hill slope and K_d_ comparing GRwt and GRdim levels were carried out using one-way ANOVA followed by Newman-Keuls post-test. Conditions with different letters are statistically different from one another (P<0.05). All results represent a minimum of two independent experiments performed in triplicate (±SEM).

COS-1 cells were transiently transfected with the established low, medium and high levels of GRwt (Fig.1A) and with GRdim. Whole-cell saturation binding assays verified that the GRdim levels obtained corresponded to the low and medium GRwt levels (Fig.2A). The receptor concentration (Bmax) and affinity (Kd) of the expressed GRs were derived from the saturation binding curves (Fig.2A), while the Hill slope was obtained from the semi-logarithmic plot of specific binding versus log M tritiated DEX (Fig. 2B).

Positive cooperative ligand-binding (Hill slope >1) was indeed observed at the higher GRwt levels, specifically at the medium and high levels (Fig.2C), confirming previous results with rat GR [43]. In contrast, the GRdim levels corresponding to the medium GRwt concentrations did not display cooperative ligand-binding (Hill slope <1), suggesting that the ability to dimerize is a requirement for cooperative ligand-binding.

Furthermore, there was a concomitant significant (P<0.05) increase in ligand-binding affinity (K_d_) of GRwt as its concentration increased which mirrors the shift to greater positive cooperative ligand-binding at the medium and high GRwt concentrations (Fig.2C). Although a similar trend occurs for the GRdim, the K_d_ at medium GRdim remained statistically similar (P>0.05) to that of low GRwt and GRdim (Fig.2C). The K_d_-value obtained in our study for high GRwt (16.8 nM) agrees with the K_d_ of 12.6 nM found in a previous study of COS-1 cells transiently transfected with high levels of GRwt [64].

These results suggest that the shift to positive cooperative ligand-binding and the associated increase in ligand-binding affinity as GRwt concentrations increase are dependent on the ability of GR to dimerize. As our Hill slopes fall between 1 and 2 this indicates binding to GR monomers as well as preformed GR dimers. Moreover, as the Hill slopes and K_d_ of the low GRwt and the low and medium GRdim concentrations were statistically similar (P>0.05) and around one, it would suggest that at low concentrations GRwt is predominantly monomeric.

### GR-dimers predominate at GRwt concentrations displaying cooperative ligand-binding

Having established a link between the ability of GR to dimerize and to bind ligand cooperatively, we sought to explore this phenomenon further using two approaches, Co-IP and FRET. As the GR forms a homodimer it was necessary to use differentially tagged GRs in order to differentiate between GR monomers and dimers.

**Figure 3:**
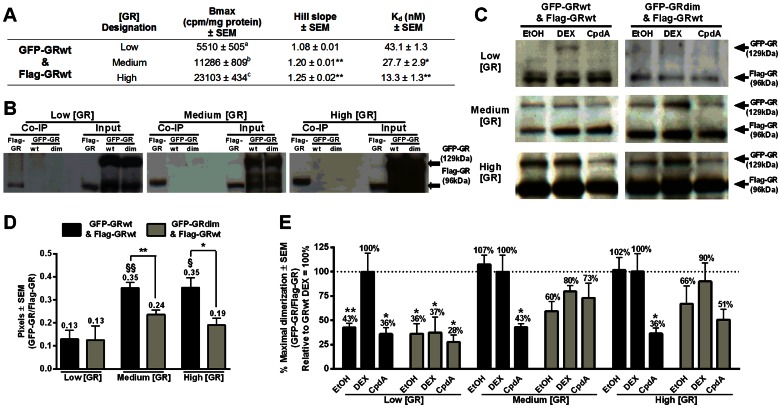
Ligand-independent dimerization of GRwt increases significantly at GR concentrations displaying cooperative ligand-binding. COS-1 cells were transiently transfected with GFP-GRwt and Flag-GRwt at low, medium or high levels. (***A***) Summary table of GFP-GRwt and Flag-GRwt saturation binding results. Statistical analysis of maximal binding (Bmax) compared to GRwt levels (Fig. 2C) was carried out using one-way ANOVA followed by Newman-Keuls post-test. Conditions with different letters are statistically different from one another (P<0.01). Statistical analysis of Hill slope and K_d_ against the low GR concentration condition were carried out using one-way ANOVA followed by Newman-Keuls post-test (P*<0.05, P**<0.01). Results represent a minimum of two independent experiments performed in triplicate (±SEM). (***B–E***) Co-IP was performed on COS-1 cells expressing low, medium or high concentrations of GFP-GRwt and Flag-GRwt or GFP-GRdim and Flag-GRwt using anti-flag beads to precipitate flag-tagged proteins. Precipitated fractions were analyzed with immunoblotting with an anti-GR antibody. Flag-GR and GFP-GR concentrations were quantified using UN-SCAN-IT software. GFP-GR pull down was then normalized over Flag-GR levels. (***B***) Co-IP controls and their inputs containing Flag-GRwt or GFP-GRwt or GFP-GRdim only at low, medium or high concentration. Cells were induced for 1 hour with 10^−6^ M DEX. (***C***) Co-IP after induction with ethanol (EtOH), 10^−6^ M DEX or 10^−5^ M CpdA for 1 hour. (***D***) Quantification of ligand-independent (EtOH) dimerization. Statistical analysis was through two tailed unpaired t tests of GRwt against GRdim (*P<0.05) and one-way ANOVA followed by Dunnett's post-test against low GR concentration within GRwt (^§^P<0.05, ^§§^P<0.01) or GRdim populations. (***E***) Normalized GFP-GR pull down, following induction with with ethanol (EtOH), 10^−6^ M DEX or 10^−5^ M CpdA, was expressed relative to DEX GRwt set at 100 percent, at each receptor level. Statistical analysis was carried out using ANOVA followed by Dunnett's post-test against DEX stimulation of GRwt at each receptor level (P*<0.05, P**<0.01). Results in (***D***) and (***E***) are representative of three independent experiments (±SEM).

We firstly utilized Flag-tagged and GFP-tagged GRwt, co-transfected and expressed at the same levels as GRwt (Fig.3A), to perform Co-IP by immunoprecipitating with anti-Flag anti-body followed by immunoblotting with anti-GR anti-body. Similarly to the GRwt (Fig.2C), this combination of differentially tagged GR's demonstrate a significant (P<0,001) shift towards greater positive cooperative ligand-binding and an increase in ligand-binding affinity at the medium and high GR concentrations (Fig.3A). Along with the pairing of Flag-GRwt and GFP-GRwt we also examined the Flag-GRwt and GFP-GRdim pair in parallel. In order to confirm that low, medium and high levels of GR were expressed, GR levels for each experiment were monitored by immunoblotting of Co-IP inputs and compared to those of the GRwt (Fig.S1A in File S1). Co-IP controls demonstrated that no non-specific pull down of either GFP-GRwt or GFP-GRdim occurred at any of the three GR concentrations studied (Fig.3B). Cells were induced with vehicle (ethanol) to test for ligand-independent dimerization (Fig. 3C). In addition, induction with the potent synthetic agonist, DEX, was used to ascertain maximal dimerization [65], while induction with the selective GR agonist, CpdA, which has previously been shown to abrogate GR dimerization [48], was used to establish minimal dimerization (Fig.3C). GFP-GR pull-down was normalized against its respective Flag-GR band in order to correct for differences in GR loading (Fig.3D&E).

There was a significant (P<0.05) increase in the level of ligand-independent dimerization at medium and high concentrations of the GFP-GRwt/Flag-GRwt pair, but not at similar concentrations of the GFP-GRdim/Flag-GRwt pair. In addition, significantly (P<0.05) lower ligand-independent dimerization via the GFP-GRdim/Flag-GRwt pair than via the GFP-GRwt/Flag-GRwt pair was observed at medium and high GR concentrations (Fig.3D). Induction of low levels of GR with DEX resulted in a significant (P<0.01) increase in GFP-GRwt pull-down, which was not observed through GFP-GRdim (Fig.3E). However, as GR levels increased, DEX-induction did not increase the level of GFP-GRwt pull-down, indicating that ligand-independent dimerization had reached its maximal level prior to DEX-induction. CpdA, which abrogates GR dimerization [48], was very effective in abolishing ligand-independent dimerization at the medium and high GR concentrations, but not at the low GFP-GRwt concentration where ligand-independent dimerization was minimal (Fig.3E). At the low concentration, GFP-GRdim displayed similar ligand-independent dimerization as GFP-GRwt, which was not appreciably affected by either DEX or CpdA addition (Fig.3E). The pull-down of GFP-GRdim showed an increase (36 to 66%) in ligand-independent dimerization as receptor levels increased from low to high GR concentration (Fig.3E). DEX addition also resulted in a trend, albeit not reaching significance, towards increased dimerization at the medium and high GRdim concentrations (Fig.3E), while CpdA addition at the high GRdim concentration resulted in a slight decrease in dimerization (Fig.3E). These results imply that in this system the GRdim is capable of some dimerization at medium and high GR concentrations. Much work has been done on this specific D-loop mutation [52], [66]-[68] and it is generally assumed to be incapable of dimerization. However, its ability to dimerize has never been tested directly until recently [69]. Our Co-IP studies, however, like those of Jewell *et al.* [69] reveal that the GFP-GRdim is capable of some ligand-independent as well as ligand-dependent dimerization to the Flag-GRwt which increases as GR concentration increases. Although GFP-GRdim dimerization is most probably enhanced by binding to the wild type Flag-GR, our results indicate that this mutation (A458T) actually results in an impairment of dimerization rather than abolition of dimerization as previously thought [70], [71]. In summation, these Co-IP results establish that at GRwt concentrations (medium and high) that result in cooperative ligand-binding (Fig.2C), ligand-independent dimerization is observed, which is not further increased by addition of DEX, but which is decreased by the addition of CpdA.

**Figure 4:**
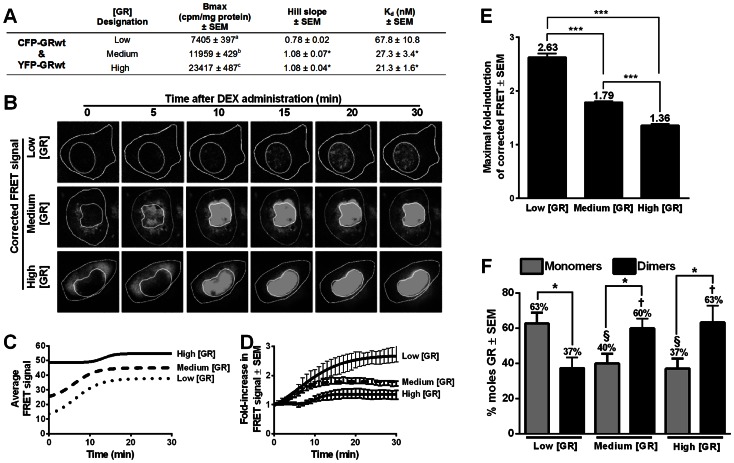
Ligand-induced dimerization decreases as GRwt concentration increases reflecting a higher percentage of ligand-independent dimerization. COS-1 cells were transiently transfected with CFP-GRwt and YFP-GRwt (low, medium or high levels). (***A***) Summary table of CFP-GRwt and YFP-GRwt saturation binding results. Statistical analysis of maximal binding (Bmax) compared to GRwt levels (Fig. 2C) was carried out using one-way ANOVA followed by Newman-Keuls post-test for Bmax. Conditions with different letters are statistically different from one another (P<0.01). For Hill slope and K_d_, Newman-Keuls post-test (P*<0.05) against the low GR concentration condition was performed. Results represent a minimum of two independent experiments performed in triplicate (±SEM). (***B–F***) FRET was carried out on cells expressing CFP-GRwt and YFP-GRwt at low, medium or high concentrations. Cells were treated with 10^-6^ M DEX for 30 minutes while F-don, F-acc and FRET was monitored at 37°C. (***B***) Representative corrected FRET signals from cells expressing CFP-GRwt and YFP-GRwt at low, medium and high levels. The outer white lines in the FRET images designate the cellular membrane which has been used as the ROI. The nucleus in each cell has also been delineated with a white line. (***C***) Average FRET signal plotted against time and fitted to a sigmoidal dose-response variable slope curve. (***D***) Fold-increase in FRET response was calculated by normalizing each experiment to its un-stimulated FRET signal and fitted to a sigmoidal dose-response variable slope curve which generates (***E***) maximal fold-induction of FRET. Statistical analysis was carried out using one-way ANOVA followed by Newman-Keuls multiple comparison test (***P<0.001). (**F**) Mathematical derivation of FRET data (supplementary information in File S1) yields percentage moles of GR occurring as either monomers or homodimers prior to ligand stimulation. Statistical analysis was through one-way ANOVA followed by Dunnett's post-test against low GR concentration within monomers (^§^P<0.05) or dimers (^†^P<0.05) and two tailed unpaired t tests comparing percentage monomers against percentage dimers at each GR concentration (*P<0.05). Results from ***C–E*** are representative of seven independent experiments (±SEM), while results in (***F***) represent a minimum of four independent experiments (±SEM).

To confirm the results obtained with Co-IP we conducted FRET experiments with CFP- and YFP-tagged GRwt. These constructs were co-transfected into COS-1 cells and were expressed at levels similar to the low, medium and high GRwt (Fig.4A & Fig.2C). In addition, a significant (P<0.05) increase in the Hill slope was also observed at medium and high GR levels (Fig.4A), similar to that evinced by GRwt (Fig.2C). The induction of FRET through CFP-GRwt and YFP-GRwt was measured before and at 1 minute intervals for 30 minutes after the addition of DEX, which induces dimerization (Fig.4B). As dimerization levels of the heterodimer pair CFP-GR and YFP-MR have been shown to be influenced by ligand concentration [72], we used 10^−6^ M DEX to induce complete dimerization as this is a saturating concentration of the ligand [55]. Cells were selected from within each population group (low, medium or high), which expressed similar levels of the two fluorescently-tagged GRs and were screened in order to ensure that the CFP-GRwt concentration within each cell fell within a predetermined range established from transfection populations used for saturation binding (Fig.S1B in File S1). The FRET images (Fig.4B) clearly show ligand-independent dimerization of GR before addition of DEX, mainly in the cytoplasm but also in the nucleus (most visible at medium and high GR concentrations), while addition of DEX results in substantial nuclear localization of dimerized GR that is fully achieved by 30 min at all concentrations of GR.

Due to the nature of our studies, direct comparison of FRET levels between the three receptor concentrations cannot be made without first normalizing for differences in GR expression. In order to correct for this, the average FRET data (Fig.4C) for each cell was normalized over its un-induced FRET value (Fig.4D). This generated curves which represent the fold-increase in FRET at each of the three GR concentrations (Fig.4D). By fitting sigmoidal dose-response variable slope curves to this normalized FRET data, maximal fold-induction data of FRET was generated for each of the three GR concentrations (Fig.4E). The levels of maximal fold-induction following DEX-stimulation decreased significantly (P<0.001) as GR concentration increased (Fig.4E). This reflects the increase in ligand-independent FRET values we see as GR concentrations increase (Fig.4B&C). In order to quantify ligand-independent dimerization we developed a mathematical model which calculates the level of GR dimerization prior to DEX administration (Supplementary model S1 in File S1). The model indicated that at low GR concentrations roughly two thirds of the receptors are monomers and that this drops to around one third monomers at the medium and high GR concentrations (Fig.4F).

The FRET results showing a significant decrease in maximal DEX-induced dimerization (Fig.4E) and a significant increase in percentage dimers prior to induction (Fig.4F) at medium and high GRwt concentrations suggest that ligand-independent dimerization occurs at these levels of GR. This is supported by the Co-IP results showing significant un-induced dimerization (Fig.3D) and maximal dimerization, which is not further increased by DEX, but is abrogated by CpdA, at these same levels of GRwt (Fig.3E). Thus together the FRET and Co-IP results suggest that at GR concentrations that show an increase in cooperative ligand-binding (Fig.2C, 3A & 4A), ligand-independent dimerization increases and is probably a prerequisite for cooperative ligand-binding.

### Transactivation of a multiple GRE-containing promoter-reporter reveals that the increase in potency and shift in the bio-character of partial agonists requires preformed GR dimers

Having established that a significant increase in the transactivation potency of the multiple GRE-containing promoter-reporter, pTAT-GRE2-E1b-luc, occurs at medium and high GRwt concentrations (Fig.1E) and that these concentrations of GRwt bind ligand cooperatively (Fig.2C) as a result of preformed GR-dimers (Fig.3D & Fig.4F) we were interested in the effect of dimerization on potency of transactivation. To this end we repeated the experiment in Fig1B and compared the effects of GRwt to that of the GRdim mutant with its diminished ability to dimerise. GR levels were once again monitored throughout by immunoblotting to ensure that low, medium and high GRwt as well as low and medium GRdim concentrations were expressed (Fig. S1A in File S1).

**Figure 5:**
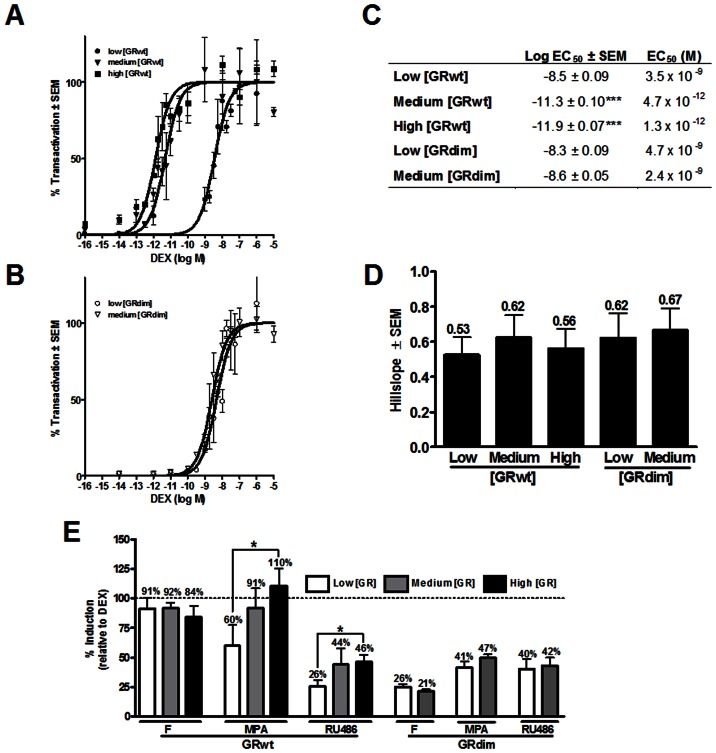
GR dimerization is required for shift in potency and bio-character in transactivation of a GRE-containing promoter-reporter. COS-1 cells were transiently transfected with (***A***) GRwt (low, medium or high levels) or (***B***) GRdim (low or medium high levels) and the GRE-containing promoter-reporter pTAT-GRE2-E1b-luc. Cells were induced with ethanol or increasing concentrations (10^−12^ to 10^−5^ M) of DEX for 24 hours. Luciferase relative light units (RLU) were normalized against protein concentrations and maximal induction to generate % transactivation data. Sigmoidal dose-response curves were fitted to the experimental data to generate (***C***) log EC_50_ and EC_50_ values. Statistical analysis of Log EC_50_ compared GRwt or GRdim to the low GRwt condition using one-way ANOVA followed by Dunnett's post-test (***P<0.001). (***D***) To generate Hill slope values for DEX transactivation, response (RLU/mg protein) was plotted against Log (M) DEX and analyzed using sigmoidal dose-response variable slope curves. (***E***) To investigate bio-character shift cells were induced with increasing concentrations (10^−12^ to 10^−5^ M) of ligand for 24 hours and dose response curves generated. Percentage induction of cortisol (F), MPA or RU486 represents maximal induction of each of these test compounds relative to that of DEX (set at 100 percent) at the same GR concentration and via the same GR construct. Statistical analysis was through two tailed unpaired t tests (*P<0.05). Results represent a minimum of three independent experiments performed in triplicate (±SEM).

Dose response curves of DEX-induced transactivation were normalised to maximal induction for easy comparison and illustrate the clear left shift in response at medium and high GRwt concentrations (Fig. 5A), while at the comparable medium GRdim level the same did not occur (Fig.5B). Statistical comparison of transactivation potency (Fig.5C) indicated significantly (P<0.001) increased potency at medium and high GRwt concentrations, but not at the medium concentration of GRdim, despite the fact that this concentration of GRdim is equivalent to that of the medium GRwt concentration (Fig.2C). In addition, GRdim in contrast to GRwt, showed no significant change in potency as its concentration increased from low to medium levels (Fig.5C). Thus there appears to be a direct correlation between increased potency in transactivation, positive cooperative ligand-binding and the presence of pre-existing GR dimers, which only occur at the medium and high GRwt concentrations. As cooperative ligand-binding is implicated in the increased potency observed at medium and high GRwt concentrations, we wondered if cooperative behaviour would also be evident for transactivation. However, no change in the Hill slope of transactivation is seen, with values at all GR concentrations reflecting non-cooperative behaviour (Fig.5D).

To establish whether the shift in potency in the transactivation of the multiple GRE-containing promoter-reporter seen with DEX at medium GRwt, but not GRdim, concentrations also holds for other ligands we induced with cortisol, the endogenous human GR ligand, MPA, a GR ligand purported to have partial agonist activity and RU486, a GR antagonist with partial agonist activity [73]. Although their potency shifts are less extreme, we saw the same trend emerging for cortisol, MPA and RU486 as for DEX (Table S2 in File S1) in that an increase in potency occurred at the medium GRwt, but not the GRdim, concentration (Table S1 in File S1). Specifically, the fold-increase in potency from low to medium GRwt concentrations for these ligands was 48-, 2.6- and 1.9-fold for cortisol, MPA and RU486, respectively, which is less than that seen with DEX and raises the question of whether positive cooperative ligand-binding, which was only demonstrated for DEX, occurs for partial agonists and antagonists. In addition the lower increase in EC_50_ seen with MPA and RU486 as a result of increased GR concentration suggests that positive cooperative ligand-binding and the potency increase it elicits may be ligand specific. Furthermore, as others had previously shown a shift in bio-character of partial GR agonists at higher GR concentrations [41], [42] we expressed maximal transactivation of cortisol, MPA and RU486 as a percentage of the maximal DEX induction at the same concentration of GRwt and GRdim (Fig.5E). The partial agonists MPA and RU486, but not the full agonist cortisol, displayed significant (P<0.05) increases in maximal efficacy relative to DEX at GRwt concentrations, which displayed positive cooperative ligand-binding due to preformed dimers (Fig.5E). Further strengthening the argument for the influence of ligand-independent dimerization on these bio-character shifts is the fact that no shift in the bio-character of either MPA or RU486 occurred as the concentration of GRdim increased (Fig.5E).

We also examined transactivation through a single GRE-containing promoter-reporter, pΔODLO (Fig.S2 in File S1), and although no significant ligand-induced activation through GRdim was observed, the transactivation efficacy generally increased with an increase in GRwt concentration (Fig.S2B in File S1), as was seen with the multiple GRE-containing promoter-reporter (Fig.1B). Ligand-independent activation also displayed a significant (P<0.001) GR concentration-dependent increase through both GRwt and GRdim, although this was significantly (P<0.01) reduced via the GRdim (Fig.S2A in File S1).

To sum up, preformed GR dimers, such as found at the medium and high GRwt, but not at the medium GRdim concentration, are required for the significant increase in the potency of transactivation of a multiple GRE-containing promoter-reporter and the shift in bio-character of partial GR agonists.

### Cooperative ligand-binding coupled to ligand-independent loading of GRwt, but not GRdim, on the endogenous GILZ gene promoter contributes to shift in potency and ligand-independent transactivation

Given that preformed GR dimers (Fig.3D & Fig.4F), with the ability to bind ligand cooperatively (Fig.2C), were shown to be necessary for the increase in potency of transactivation of a synthetic GRE-containing promoter-reporter (Fig.5C) and that ligand-independent transactivation of the same promoter was shown to increase exponentially with increased GRwt concentrations (Fig.1C), we sought to test these parameters on the endogenous GILZ gene. The GILZ protein is a potent anti-inflammatory regulatory protein [74], the promoter of which is known to contain multiple GRE's [75] shown to be under the direct control of activated GR [56], [76].

**Figure 6:**
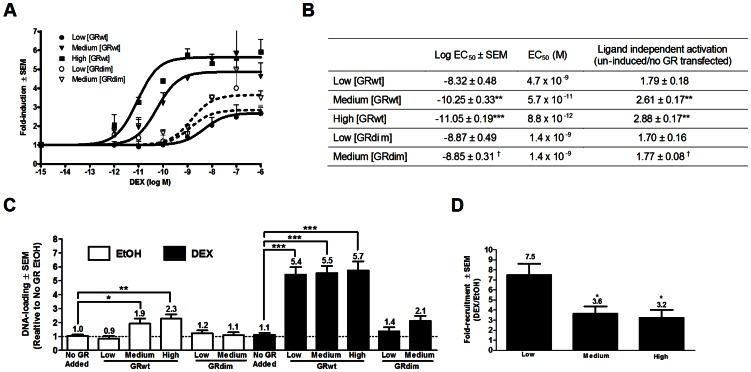
Dimerization of GR is essential for ligand-independent loading of GR on endogenous GILZ gene promoter. (***A–B***) COS-1 cells expressing GRwt (low, medium or high levels) or GRdim (low and medium levels) were induced for 8 hours with either ethanol or a range of DEX concentrations. RT-PCR of the GILZ gene was conducted and expression calculated relative to GAPDH for each condition. Sigmoidal dose-response curves were fitted to the experimental data. (***A***) Dose response curves of fold-induction of GILZ gene. (***B***) Summary table of GILZ transactivation results. Ligand-independent activation of GILZ is expressed relative to cells with no transfected GR. Statistical analysis of GILZ transactivation parameters comparing GRwt or GRdim to the low GRwt condition were carried out using one-way ANOVA followed by Dunnett's post-test (**P<0.01, ***P<0.001) and two tailed unpaired t tests of low GRwt against low GRdim or medium GRwt against medium GRdim (^†^P<0.05). (***C–D***) COS-1 cells transfected without or with GRwt (low, medium or high levels) or GRdim (low and medium levels) were induced for 1 hour with either ethanol or 10^−6^ M DEX. ChIP was performed using anti-GR antibody. RT-PCR of the promoter of the GILZ gene was performed on input chromatin, as well as the chromatin precipitated with anti-GR antibody. GILZ pull-down was normalized to input DNA. (***C***) GR recruitment to GILZ promoter expressed relative to the ethanol-induced no GR transfection condition. (***D***) DEX-induced fold-recruitment of GRwt to the GILZ promoter expressed relative to the ethanol condition within each transfection group. Statistical analysis of DNA-loading (***C***) comparing either ethanol- or DEX-induced conditions to their respective no GR transfected condition were carried out using one-way ANOVA followed by Dunnett's post-test (*P<0.05,**P<0.01, ***P<0.001). Fold-recruitment following DEX induction (***D***) statistical analysis comparing to low GRwt levels was through one-way ANOVA followed by Dunnett's post-test (*P<0.05). All results represent a minimum of three independent experiments performed in triplicate (±SEM).

As before COS-1 cells were transiently transfected with low, medium and high GRwt or low and medium GRdim levels and GR expression levels were monitored by immunoblotting (Fig.S1A in File S1). Cells were induced with a range of DEX concentrations and GILZ mRNA expression plotted relative to un-induced vehicle (ethanol) levels (Fig.6A). Similarly to the promoter-reporter assay (Fig.5A&B), a left shift in dose response curves was seen with increased concentrations of GRwt, but not with GRdim (Fig. 6A). At medium and high GRwt concentrations, but not at medium GRdim concentrations, a significant (P<0.01) increase in the potency of transactivation of the GILZ gene was observed (Fig.6B). Specifically, at medium GRwt concentrations an 80-fold increase was observed, which escalated to a 500-fold increase at high GRwt levels. Furthermore, although no significant (P>0.05) difference was seen between the potency values at low GRwt and GRdim levels, at medium GR levels there was a significant (P<0.05) 25-fold difference between the GRwt and GRdim (Fig.6B). In addition, ligand-independent transactivation increased significantly (P<0.01) as GRwt concentration increased, but not as GRdim concentration increased (Fig.6B). Moreover, the ligand-independent transactivation at the medium concentration of GRwt was significantly (P<0.05) greater than that at the medium concentration of GRdim (Fig.6B), highlighting the importance of dimerization in ligand-independent transactivation.

The increase in ligand-independent transactivation at GRwt concentrations shown to have increased ligand-independent dimerization (Fig.3D & Fig.4F), coupled to the fact that the magnitude of the increase in transactivation potency of 500-fold at high GRwt levels appears to exceed the magnitude expected from a 4-fold increase in GR levels and a 3-fold increase in ligand-binding affinity (Fig.2C) suggested that additional factors may be involved in the increase in potency. As binding of the GR to DNA is a primary requirement [77], [78] for transactivation we examined loading of the GR onto the promoter of the GILZ gene. COS-1 cells were transfected with GRwt (low, medium and high levels) and GRdim (low and medium levels) and induced with vehicle and DEX (10^−6^ M) before ChIP was performed using the GR antibody. Ligand-independent recruitment to the promoter was evaluated by comparing loading after ethanol induction in the absence of transfected GR to loading in the presence of the transfected GR (Fig.6C, open bars). A significant (P<0.05) 2-fold increase in loading of medium and high concentrations of GRwt was observed, while no increase in loading was observed at medium GRdim concentrations despite the fact that the GR concentrations are not significantly (P>0.05) different from that of medium GRwt (Fig.2C). It may be argued that an increase in DNA loading reflects the increased GR pull-down at medium and high GRwt levels, however, the low GRwt concentration displayed no increase in non-specific DNA-loading when compared to the no GR transfected condition and neither did the low or medium GRdim concentrations (Fig.6C). In the presence of saturating concentrations of DEX, however, similar loading of GRwt resulted (Fig.6C, closed bars). Pursuant to this, calculation of the DEX-induced fold-recruitment of GRwt (Fig.6D) showed a significantly (P<0.01) increased loading at low GRwt concentration, due to the significantly (P<0.05) lower basal, ligand-independent loading of the GRwt (Fig.6C, open bars).

To conclude these results suggest that the ability of GRwt, but not GRdim, to form ligand-independent dimers is a prerequisite for cooperative ligand-binding and priming of the GILZ promoter by ligand-independent DNA-loading of GRwt, which facilitates ligand-independent transactivation and an increase in the potency of transactivation.

## Discussion

This study aimed to evaluate and quantify the impact of GR concentration on GC sensitivity. We now report that at high receptor density the GRwt undergoes ligand-independent dimerization, which results in cooperative ligand-binding and basal priming of transactivation, both of which produce a significant increase in transactivation potency as well as a shift in the bio-character of partial agonists. Furthermore, we show that if the ability to form ligand-independent GR dimers is abrogated this behaviour is attenuated.

We show that at higher (>153 fmol/mg protein) concentrations of GRwt ligand-independent dimerization of the GR increases significantly. Specifically, CoIP and FRET studies in living cells indicate a 2- to 3-fold increase in ligand-independent dimerization at these higher GRwt concentrations, which is not obtained with similar concentrations of the dimerization deficient mutant, GRdim (Fig.3D & Fig.4F). In the absence of ligand these preformed GR-dimers are shown to have two immediate down-stream effects. Firstly, a 2-fold increase in ligand-independent GRwt loading on the GILZ promoter (Fig.6C), and secondly, a 2 or 10-fold increase in ligand-independent transactivation of the GILZ gene (Fig.6B) or GRE-containing promoter-reporters (Fig. 1C & Fig.S2A in File S1), respectively. The fact that at similar concentrations the GRdim did not result in significant ligand-independent loading of the GR onto the GILZ promoter (Fig.6C), nor did it produce comparable ligand-independent transactivation (Fig.6C & Fig.S2A in File S1) substantiates the view that preformed GR dimers are required for this behaviour. In the presence of ligand the preformed GRwt dimers, resulting from higher GR concentrations, show cooperative ligand-binding (Hill slope>1) and display up to a 3-fold increase in ligand-binding affinity (1/Kd) for DEX (Fig.2C). Pursuant to ligand-independent loading of GR onto promoter and cooperative ligand-binding the potency (EC_50_) of DEX transactivation increases significantly, up to 2600-fold for the multiple GRE-containing promoter-reporter (Fig.5C) and up to 500-fold for the GILZ gene (Fig.6B). Furthermore, preformed GRwt dimers potentiate the shift in bio-character of partial agonists, such as MPA and RU486 (Fig.5E). Neither cooperative ligand-binding nor the increase in transactivation potency or bio-character shift of partial agonists is observed when GRdim is used at a higher concentration, which supports our claim that preformed GR dimers are responsible for these phenomena.

### Model

**Figure 7:**
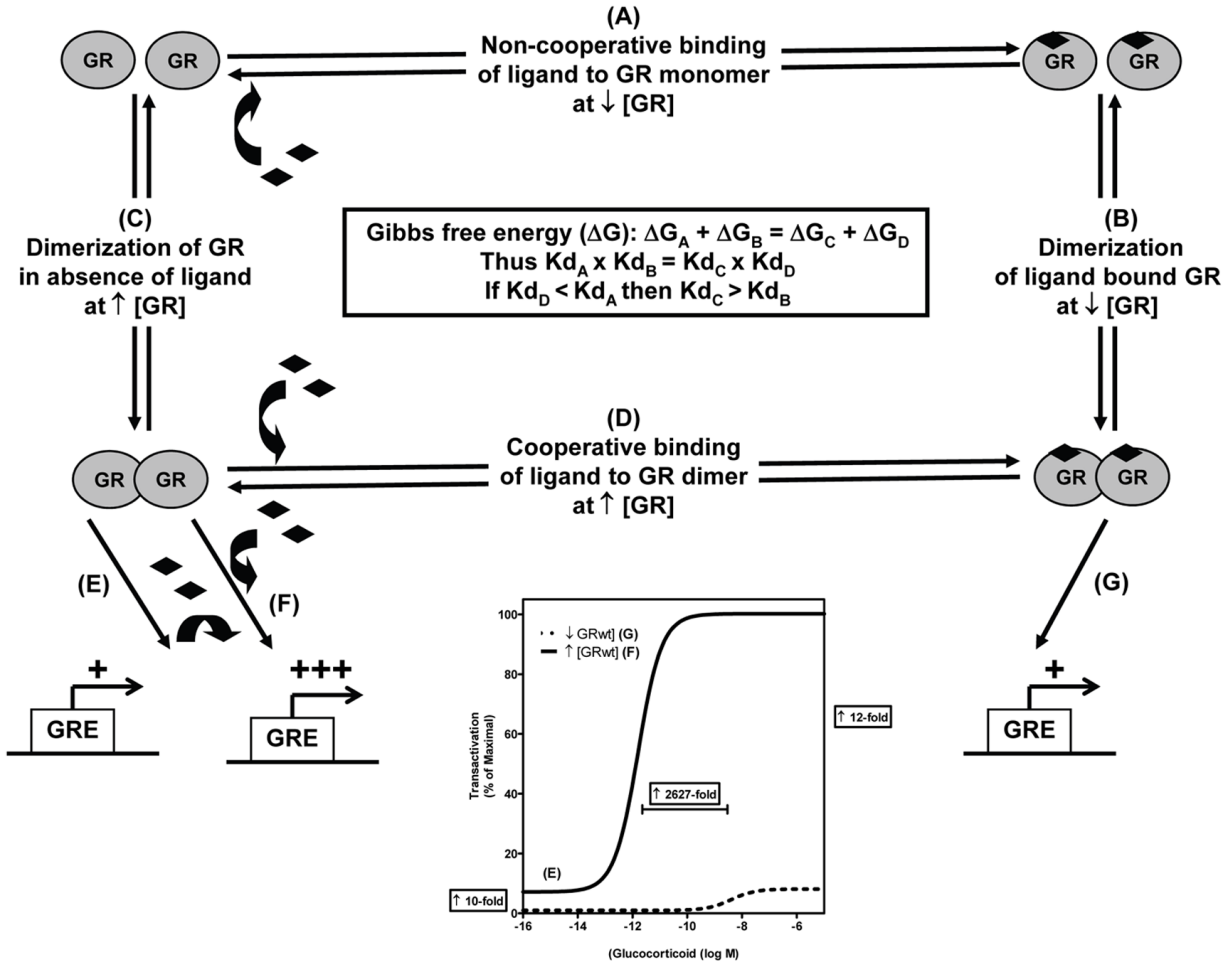
Model comparing down-stream effects at high and low GRwt concentrations. Pathways (***A***), (***B***) and (***G***) denote conditions at low GR concentrations. (***A***) Non-cooperative ligand-binding to GR monomers, followed by (***B***) ligand-dependent dimerization and (***G***) transactivation. Pathways (***C***), (***D***), (***E***) and (***F***) denote conditions at high GR concentrations. (***C***) Ligand-independent dimerization of the GR, followed by either (***E***) ligand-independent DNA loading and transactivation and/or (***D***) cooperative ligand-binding and (***F***) transactivation with increased potency. **Graph insert**: Transactivation results from Fig. 1B reworked as percentage maximal transactivation. Indicated is fold-increase in ligand-independent transactivation (***E***) and increase in transactivation efficacy and potency at higher concentrations of GR - difference between (***G***) and (***F***).

Our results suggest two parallel mechanisms for GR signalling governed by GR concentration (Fig.7). At low concentrations of GR, monomeric GR predominates and non-cooperative ligand-binding (Fig.7A) occurs, which then potentiates ligand-induced dimerization (Fig.7B). In contrast, at high GR concentrations the monomer-dimer equilibrium shifts and more dimers are present (Fig.7C). These preformed, ligand-independent dimers display positive cooperative ligand-binding (Hill slope>1), which implies an increase in ligand-binding affinity (Fig.7D). Thermodynamically, the model is supported as the free energy (ΔG) to produce ligand-bound dimerized GR is the same, whether proceeding via preformed GR dimers or via ligand-induced dimerization (Fig.7 insert).

Once ligand-independent dimerization has occurred our model suggests that association with DNA can occur and result in an increase in basal transactivation (Fig.7E). Pursuant to ligand-independent loading of GR to promoter or priming of the promoter, added ligand will now result in a response with increased potency (Fig.7F). In contrast at low GR levels where priming does not occur, as GR monomers predominate in the unliganded state, addition of ligand produces a response with a lower potency (Fig.7G)

### GR dimerization

Intrinsic to our model concerning GR dimerization are the presumptions (i) that the GR can dimerize in the absence of ligand and (ii) that GR can dimerize in the absence of DNA or (iii) that unliganded, but dimerized, GR can bind DNA, albeit with lower affinity than liganded, dimerized GR.

### Ligand-independent dimerization

Few researchers have tackled the first presumption, that of ligand-independent dimerization at high GR concentrations, directly and furthermore their results are contradictory. One of the few findings that directly support our CoIP (Fig.3D) and FRET results (Fig.4F), indicating ligand-independent dimerization at high GR concentrations, is that by Drouin *et al.* [79] who diluted purified rat liver GR to shift the equilibrium of GR from homo-dimers present in the concentrated GR preparation to monomers in the diluted preparation. Unfortunately, no direct evidence for this is provided in the published work. However, Savory *et al*. [65] using high salt conditions to dissociate immunoprecipitated rat GR from its chaperone complex also demonstrate ligand-independent dimerization of the GR in the cytoplasm, although they did not investigate the effect of GR concentration on this phenomenon. In contrast, although Robblee *et al*. [80], using purified human GR in the absence of ligand and analytical ultracentrifugation, observe a species consistent with a GR dimer, they conclude that it reflects a functionally incompetent species as its concentration (∼7%) does not increase with increased GR levels. Wrange *et al*. [81], however, found that when using glycerol gradient centrifugation of purified rat GR, ligand-bound GR-dimers were unstable unless stabilised by either DNA-binding or glutaraldehyde cross-linking but when gel filtration was used the dimer was stable. Furthermore, they also found, using glycerol gradient centrifugation, that only the preformed GR-dimer-DNA complex was stable in the absence of ligand. Taken together the results from Robblee and Wrange may suggest that the unliganded GR dimer is much less stable than the ligand-bound dimer and that CoIP, such as used in the present study, rather than centrifugation may be a better technique to observe this GR species. Furthermore this would further support our model by suggesting that the K_d_ for the dimerization of unliganded GR (Kd_C_ in Fig.7) is greater than that of ligand-bound GR (Kd_B_ in Fig.7).

### Ligand-dependent dimerization without DNA

The second presumption that GR can dimerize in the absence of DNA has most often been addressed in the literature by re-phrasing the question as whether GR (ligand-bound in most instances) binds to GREs as a preformed dimer or dimerizes following binding of two GR monomers to each of the two half-sites within the GRE. Here again the literature is controversial. Our FRET results (Fig.4B) certainly confirm that GR dimerization can occur in the cytoplasm and is therefore independent of DNA binding. This finding is supported by immunoprecipitation [65] and centrifugation [81] studies demonstrating association of preformed ligand-induced GR-dimers to DNA. Robblee *et al*. [80] refute these findings by suggesting that ligand-bound GR is exclusively monomeric and that dimerization only occurs due to cooperative binding of the GR-monomers to DNA, however, as discussed above it may be that the ultracentrifugation technique used in this study is not ideal for observing the GR-dimer. Ong *et al.* [82] also strongly oppose the idea of pre-formed GR-dimers binding to DNA, using both a theoretical (based on the fact that multiple steps in the GR-signal transduction cascade preserve a first-order Hill dose–response curve (FHDC), which cannot theoretically be maintained if preformed dimers bind DNA) and an experimental approach (showing that GRdim mutants maintain a FHDC). Although DNA-independent GR dimerization does not preclude the binding of GR monomers to the DNA and their subsequent dimerization, it would suggest that were GR dimerization to be abrogated (by using GRdim mutants, for example) it should lead to an increase in potency due to the increased availability of monomers. Tellingly, however, results from Ong *et al.* [82], as well as our own results (Fig.5C & Fig.6B), show far greater potency through the GRwt than via the GRdim.

### Ligand-independent dimers can bind to DNA

Further support not only for the binding of pre-formed GR dimers, but also of unliganded GR dimers, to DNA comes from electro mobility shift studies [79] showing not only that unliganded GR can bind to DNA but that the affinity of the GR-monomer is much lower than that of the GR-dimer (K_d_ = 62 nM versus K_d_ = 1.21 nM [77] or 1nM versus 0.23nM [79]). Association studies comparing the association of unliganded GR dimer to that of GR monomer then also show slower kinetics for the binding of GR monomer to DNA [79]. This then supports our ChIP results showing that unliganded GR dimers, but not GR monomers, load onto DNA (Fig.6C). Further support for the presence of unliganded GR dimers in the nucleus comes from our FRET results (Fig.4B) that show the presence of GR dimers in the nucleus, albeit at lower levels than in the cytoplasm, before addition of ligand at medium and high GR concentrations. Intriguingly, not-withstanding their assertion that this is via sequential monomer association, the work by Robblee *et al*. [80] suggests that in the presence of ligand the total binding affinity for saturating a single DNA response element with two GR monomers (K_a_ = 5.18×10^13^ M^−2^) is higher than that found in the absence of ligand by Drouin [79] or Segrad-Maurel [77]. Together these data suggest a hierarchy of binding affinities for the GR: ligand-bound, dimerized GR > unliganded, dimerized GR ≥ ligand-bound, monomeric GR > unliganded, monomeric GR which is consistent with our model ((E) & (F/G) in Fig.7) and ChIP results (Fig.6C).

### Cooperative ligand-binding

Theoretically, positive cooperative ligand-binding implies ligand-independent dimerization of the GR, which effectively creates two ligand-binding sites where the association of the first ligand facilitates the binding of the second in a more energetically favourable reaction and thus increases the affinity of the receptor for the ligand [83]. The Hill slope is known to provide a minimum estimate of the number of binding sites involved [62] with a theoretical maximum Hill slope for binding to a receptor with a single binding site of 1, which increases to a maximum of 2 when two binding sites are present. Our results demonstrated positive cooperative ligand-binding to the GRwt as GR concentration increased 4-fold (Fig.2C) with Hill slope values for untagged-GRwt increasing up to 1.72 (Fig.2C), which closely matches *in vitro* binding studies demonstrating a shift in Hill slope from 1.0 to 1.5 as GRwt concentrations increased 4-fold [43]. Furthermore, the increase in Hillslope from 1.08 to 1.72 represents a 6-fold decrease in the concentration of ligand required to shift receptor occupancy from 10 to 90% and a 3-fold increase in ligand-binding affinity (1/K_d_) (Fig.2C). The canonical view of ligand-binding affinity has been seen as “an invariant parameter across tissues within a species” [1], however, there is mounting evidence that a variety of factors may influence this parameter. The phosphorylation state of the GR [84], the presence of proteins affecting the free concentration of GCs such as CBG [85] and 11βHSD [11], and co-modulators of the receptor such as Hsp90 [86], immunophilins (FKBP52 and PP5) [87] and ubiquitin-congugating enzyme 9 (Ubc9) [43] have all been shown to alter ligand-binding affinity. In addition, in a study investigating the GR levels in female Sencar mice treated with 12-*O*-tetradecanoylphorbol-13-acetate (TPA) a 4-fold reduction in GR levels was accompanied by a 2- to 4-fold increase in K_d_ [22], supporting our results (Fig.2C) and substantiating our model (Kd_A_ > Kd_D_ in Fig.7). Furthermore, although dimerization of the GR [43] and ER [88] has been linked to its ability to bind ligand cooperatively at high concentrations, our results are the first to correlate this behaviour with an increase in ligand-independent dimerization (Fig.3 & Fig.4). Since positive cooperative ligand-binding implies ligand-binding to more than one binding site, our results strongly suggest that ligand-independent dimerization at high concentrations of GRwt facilitates positive cooperative ligand-binding [83]. This is supported by the fact that GRdim was unable to elicit cooperative ligand-binding at higher GR concentrations (Fig.2C) and further corroborates our model predicting preformed GRwt dimers at higher concentrations (compare (A) to (D) in Fig.7).

Ligand-independent dimerization of the GR may not only facilitate cooperative ligand-binding due to the creation of two ligand-binding sites but may result in a receptor conformation which results in the recruitment of co-modulators which enhance ligand-binding affinity. It follows that a dynamic ligand-binding affinity is also linked to the receptor's ability to dimerize and may be an evolutionary mechanism whereby tissue specific GC sensitivity is achieved.

### Down-stream effects on transactivation and physiological relevance

Having elucidated the immediate effects of ligand-independent dimerization of the GR at high concentrations, namely, ligand-independent DNA-loading and cooperative ligand-binding, we tested its down-stream consequences on ligand-independent and ligand-dependent transactivation. The effect of ligand-independent GR loading of preformed GR-dimers on the GILZ promoter is evidenced through our RT-PCR results, which demonstrate significantly increased ligand-independent transactivation of the GILZ gene at higher GRwt, but not GRdim, concentrations (Fig.6B). Furthermore, this increase in basal transactivation also seen with the GRE-containing promoter-reporters (Fig.1C & Fig.S2A in File S1) is similar to reports in the literature [89]. Intriguingly, since several recent reports have shown that the GR, in response to a variety of effectors, can be transcriptionally activated and recruited to endogenous promoters in the absence of GCs [1], [90], [91], [91]–[94,94], it is tempting to speculate that this may involve increased GR loading of preformed GR-dimers onto the DNA. While ligand-independent activation of the GR may be especially relevant to the treatment of inflammatory diseases, the mechanisms may not always involve ligand-independent GR promoter recruitment [1], [95]. However, Bain *et al*. [78] suggests that the interaction of GR with DNA may be the primary determinant of transcriptional activity, consistent with our model (see (E) leading to (F) in Fig.7).

Our model suggests that pursuant to increased GRwt concentrations, ligand-independent dimers form, which results in ligand-independent DNA-loading and basal transactivation. Addition of ligand, which can then bind cooperatively to these preformed and preloaded GR-dimers, causes an exponential increase in the potency (EC_50_) of transactivation (see (F) in comparison to (G) in Fig.7). Our results both on a GRE-containing promoter-reporter (Fig.1E & Fig.5C) and an endogenous gene containing GREs in its promoter (Fig.6B) certainly show a significant increase in potency at higher GRwt, but not GRdim, concentrations corroborating the model (see (F) versus (G) in Fig.7). While the influence of GR concentration has not been examined on an endogenous gene before, a number of studies have demonstrated an increase in the transactivation potency at increased GRwt concentrations in promoter reporter assays [39], [43]. However, the only article which calculated an EC_50_ for transactivation only demonstrated a 7.5-fold increase in potency at an unspecified increase in GRwt concentration [43]. Intriguingly, the same article alludes to the fact that the GR coactivator, ubiquitin-congugating enzyme 9 (Ubc9), influences high GRwt concentrations preferentially resulting in a further 5.1-fold increase in potency when co-expressed [43]. Indeed, even the combination of increased GRwt concentration and Ubc9 co-expression may only result in a 38-fold increase in potency. This is a far cry from our findings which are hard to explain simply through the marginal increases in cooperative ligand-binding and ligand-binding affinity. Yet the introduction of ligand-independent DNA-loading is a powerful mechanistic tool, which may encompass the priming of ligand-dependent transcription. As has recently been revealed, the activated GR binds almost exclusively to accessible chromatin [96], which is rendered as such by ancillary transcription factors, most notably the activator protein 1 (AP1) [97]. Priming of GR-responsive chromatin by the binding of AP1 is a prerequisite for maximal GR recruitment and transcription. We thus hypothesize that GR itself may also, like AP1, act as a pioneering factor through ligand-independent DNA-loading of GR at increased GRwt concentrations and may act in a similar fashion as AP1to recruit coactivators and comodulators independently of ligand-binding, which when coupled to increased GR concentration as well as cooperative ligand-binding may account for the magnitude of the increase in the potency of transactivation observed in our studies.

In a recent article from this group [98] we examined the influence of GR-concentration and the ability to dimerize on the rate of nuclear import and export as well as nuclear localization. The same receptor concentrations were used as in the current study, and although we found a reduction in the observed rate of nuclear import and increased rate of nuclear export at low GRwt concentrations and by the GRdim, the results did not, however, show that cooperative ligand-binding, and thus presumably the formation of ligand-independent dimers, influenced the examined parameters. Thus we suggest that longer nuclear retention with faster kinetics of import may be GR-concentration dependent rather than being influenced by the formation of preformed GR-dimers.

As has been demonstrated for DEX-Mes, RU486 and Prog in promoter-reporter assays [18], [41], we also show that increasing GRwt levels result in a bio-character shift of the partial agonists MPA and RU486, (Fig. 5E). Newton *et al.* [1], in a recent review, provides a very convincing explanation for the bio-character shift of partial GR agonists at higher GR levels. However, in our work the shift from weak to partial agonist or from partial to full agonist for RU486 and MPA, respectively, at higher GR levels is not seen with GRdim (Fig. 5E). Thus we suggest that the observed bio-character shift is not only due to increased GR levels as suggested by Newton [1] but are directly linked to preformed GR-dimers and cooperative ligand-binding. Although cooperative ligand-binding has only been demonstrated for DEX, these results (Fig. 5E) suggest that the mechanistic change facilitated by ligand-independent dimerization, which has been demonstrated at the GRwt levels concerned, may alter the behaviour of partial agonists, enhancing their capacity to transactivative. Furthermore, the fact that GRdim does not elicit this bio-character shift at higher GR concentrations further supports the concept that preformed GR dimers, at higher GRwt concentrations, are a prerequisite for the shift in bio-character of partial agonists.

The physiological relevance of this massive increase in potency at GR concentrations which display ligand-independent dimerization is that in tissues with high enough GR concentrations, genes with GRE-containing promoters will be maximally activated by even trace concentrations of GC. Therefore, even the lowest levels of endogenous or pharmacologically administered GC will be enough to induce maximal induction in tissues with high enough GR concentrations. This increase in GC sensitivity has been elegantly demonstrated *in vivo* in two gain of function knock-in mouse models [99], [100]. Furthermore, these tissues will have lost the capacity to regulate their response to alterations in GC levels. To illustrate, the concentration of free cortisol in the blood varies from ∼18.7 nM in the morning to ∼3.3 nM at night [101], which would mean that the increased EC_50_ value from 1.7 nM at low GRwt concentrations to 0.03 nM at medium GRwt concentrations for F (Table S1 in File S1) would entail a shift from a varying circadian influence of endogenous cortisol on cells expressing GR at the low concentration to a maximal response in cells expressing the medium GR concentration.

Cells with GR concentrations high enough to result in the significant formation of preformed ligand-independent dimers, displaying positive cooperative ligand-binding and ligand-independent priming of DNA, would therefore be hypersensitive to DEX and would exist in a state of maximal transactivative response once exposed to ligand were it not for ligand-induced down-regulation of the GR. It is important to stress that the medium to high GRwt concentrations reflect a GR concentration range of 153 to 284 fmol GR/mg protein and although some tissues do express GR at these elevated levels [28], the majority of healthy cells within the human body will retain the capacity to respond to changes in GC concentration due to their relatively low GR levels. In addition, as the t_½_ of unstimulated GR following incubation with cycloheximide has been shown to be 44 hours, which drops to 10 hours following stimulation with 10^-5^ M DEX and cycloheximide [102], down-regulation of the GR would eventually result in GR levels low enough to no longer result in positive cooperative ligand-binding, although this would probably take longer than the t_½_ quoted in a system that was not exposed to cycloheximide. Although a decrease in GR concentration would blunt the hypersensitivity of cells expressing higher GR concentrations, our results clearly indicate that this blunting of response has not occurred following 24 hours of DEX exposure. Furthermore, it has been demonstrated in binding assays conducted on human tissue biopsies that elevated GR levels are maintained despite exposure to physiologically basal GC levels [28], [58].

### Functional diversity elicited by dimerization

The interactions between proteins form part of nearly all biological processes. Dynamic dimerization refers to the transitory, non-covalent, association of two identical or closely related proteins in response to a particular signal [103], [104]. More often than not dimerization results in an active complex the formation of which initiates a signalling process, such as seen in the nuclear receptor family [65], [105]. Furthermore, the advantage of closer proximity and favourable orientation elicited by GR dimerization may account for the increase in DNA-binding affinity displayed by GR dimers [77]. Intriguingly, the process of dimerization itself is sufficient to activate some receptors [106], [107], and has been shown to increase as receptor concentration increases [88]. Thus the ligand-dependent as well as ligand-independent dimerization of receptors may be viewed as an evolutionary mechanism through which greater functional diversity can be elicited through a single protein species.

## Conclusion

Considering the ubiquity of dynamic dimerization as a tool for enhancing the functional diversity of proteins and nuclear receptors in particular, could it be that ligand-independent dimerization of the GR at high concentrations is a mechanism employed physiologically to impart hypersensitivity to cells expressing high GR levels?

Taken together our results suggest that cells containing higher GR levels are primed, through DNA-loading of preformed GR dimers, to respond to GCs at much lower concentrations due to the increased affinity and cooperative ligand-binding brought about by ligand-independent dimerization of the GR. This may help to explain differences in tissue specific responses to GCs and garner insight into GC hyposensitivity and hypersensitivity disorders. Clearly GR concentration has far ranging effects on the response to GCs and must be taken into account when designing and comparing tissue culture experiments, the staple of most pharmacological research.

## Supporting Information

File S1 **Figure S1. Whole-cell saturation binding, immunoblotting and fluorescent intensity used to monitor and determine GR levels.** (*A*) COS-1 cells were transfected with GRwt or GRdim (low, medium or high levels) during assays. Immunoblotting was performed (see Material and Methods) on cell lysates and pixels from densitometric analysis of the immunoblots was correlated to GR levels (cpm/mg protein) determined by whole cell saturation binding (see Materials and Methods). A standard curve correlating GR concentrations in cpm/mg protein derived from saturation binding to their respective densitometric values (pixels) from immunoblotting was produced (R^2^ = 0.9719). This curve was used to monitor and determine GR levels throughout. (*B*) For FRET assays the relative CFP-GR (F-don) expression levels in individual cells within low, medium and high GR concentration populations were measured and used to monitor GR levels. Exposure times of 1500 ms at 100% light intensity were used. F-don values reflect the CFP signal after 30 minutes of DEX stimulation measured in a region of interest in the nucleus of each individual cell. Cells with an F-don emission of 0–600 where selected for the low [GR] concentration (_*_, n = 10), F-don signals between 600–1200 for the medium [GR] population (†, n = 7) and F-don of >1200 for the high [GR] population (§, n = 7).Figure S2. Un-induced transactivation increases and fold-induction decrease at higher GRwt concentration through single GRE. Cells were transfected with GRwt or GRdim (low or medium levels) and 3000 ng pΔODLO, a promoter-reporter containing a single GRE. Cells were induced with ethanol, 10^−6^ M DEX, F, MPA or RU486 for 24 hours. Luciferase activity was determined and relative light units (RLU) were normalized against protein concentrations. (*A*) Un-induced RLU/mg protein values following 24 hours ethanol stimulation. Statistical analysis was through two tailed unpaired t tests of low GRwt concentration against medium GRwt concentration (^†††^P<0.001), low GRdim concentration against medium GRdim concentration (^§§§^P<0.001) and GRwt against GRdim (**P<0.01, ***P<0.001). (*B*) Maximal induction and (*C*) fold-induction (calculated as maximal induction normalized to un-induced induction) were plotted. Statistical analysis was through one-way ANOVA followed by Dunnett's post-test comparing un-induced (ethanol) conditions to the ligand-induced conditions within the low (^†††^P<0.001) or medium (^§^P<0.05, ^§§^P<0.05) concentration populations of GRwt or GRdim and two tailed unpaired t tests of ligand-induced low GRwt concentration against medium GRwt concentration (*P<0.05, **P<0.01, ***P<0.001). All results represent two experiments performed in triplicate (±SEM). Model S1. Mathematical model to calculate percentage monomers from FRET data. Table S1. Average GR concentrations per cell at each GR concentration. Table S2. GR levels and the ability to dimerize influences potency (log EC_50_) of transactivation in a range of ligands. Cells were transfected with GRwt or GRdim (low or medium levels) and pTAT-GRE2-Elb-luc. Cells were induced with ethanol or a range (10^−12^ M to 10^−5^ M) of F, MPA, or RU486 for 24 h. Luciferase activity was determined and relative light units normalized against protein concentrations. Sigmoidal dose-response curves where fitted to the experimental data which generated the potency (Log EC_50_), maximal induction (Bmax) and fold-induction. Statistical analysis was carried out on logEC_50_-values using one-way ANOVA followed by Newman-Keuls post-test: (*P<0.05, **P<0.01, ***P<0.001) to compare GRwt and GRdim to the low GRwt condition and (^†^P<0.05, ^††^P<0.01, ^†††^P<0.001) to compare low GRwt against low GRdim or medium GRwt against medium GRdim. All results represent a minimum of three independent experiments performed in triplicate (±SEM).
(DOC)
