# Supplementary material for: Impact of Glucocorticoid Receptor Density on Ligand-Independent Dimerization, Cooperative Ligand-Binding and Basal Priming of Transactivation: A Cell Culture Model
Source: PLoS One. 2013 May 22;8(5):e64831. doi: 10.1371/journal.pone.0064831 (PMC3661511; doi:10.1371/journal.pone.0064831)
Supplement: File S1 — Figure S1. Whole-cell saturation binding, immunoblotting and fluorescent intensity used to monitor and determine GR levels. (A) COS-1 cells were transfected with GRwt or GRdim (low, medium or high levels) during assays. Immunoblotting was performed (see Material and Methods) on cell lysates and pixels from densitometric analysis of the immunoblots was correlated to GR levels (cpm/mg protein) determined by whole cell saturation binding (see Materials and Methods). A standard curve correlating GR concentrations in cpm/mg protein derived from saturation binding to their respective densitometric values (pixels) from immunoblotting was produced (R2 = 0.9719). This curve was used to monitor and determine GR levels throughout. (B) For FRET assays the relative CFP-GR (F-don) expression levels in individual cells within low, medium and high GR concentration populations were measured and used to monitor GR levels. Exposure times of 1500 ms at 100% light intensity were used. F-don values reflect the CFP signal after 30 minutes of DEX stimulation measured in a region of interest in the nucleus of each individual cell. Cells with an F-don emission of 0–600 where selected for the low [GR] concentration (*, n = 10), F-don signals between 600–1200 for the medium [GR] population (†, n = 7) and F-don of >1200 for the high [GR] population (§, n = 7).Figure S2. Un-induced transactivation increases and fold-induction decrease at higher GRwt concentration through single GRE. Cells were transfected with GRwt or GRdim (low or medium levels) and 3000 ng pΔODLO, a promoter-reporter containing a single GRE. Cells were induced with ethanol, 10−6 M DEX, F, MPA or RU486 for 24 hours. Luciferase activity was determined and relative light units (RLU) were normalized against protein concentrations. (A) Un-induced RLU/mg protein values following 24 hours ethanol stimulation. Statistical analysis was through two tailed unpaired t tests of low GRwt concentration against medium GRwt concentration (†††P [file pone.0064831.s001.doc]

**Supporting Information:**

**Figure S1.** **Whole-cell saturation binding, immunoblotting and fluorescent intensity used to monitor and determine GR levels. (*A*)** COS-1 cells were transfected with GRwt or GRdim (low, medium or high levels) during assays. Immunoblotting was performed (see Material and Methods) on cell lysates and pixels from densitometric analysis of the immunoblots was correlated to GR levels (cpm/mg protein) determined by whole cell saturation binding (see Materials and Methods). A standard curve correlating GR concentrations in cpm/mg protein derived from saturation binding to their respective densitometric values (pixels) from immunoblotting was produced (R^2^=0.9719). This curve was used to monitor and determine GR levels throughout. (***B***) For FRET assays the relative CFP-GR (F-don) expression levels in individual cells within low, medium and high GR concentration populations were measured and used to monitor GR levels. Exposure times of 1500ms at 100% light intensity were used. F-don values reflect the CFP signal after 30 minutes of DEX stimulation measured in a region of interest in the nucleus of each individual cell. Cells with an F-don emission of 0-600 where selected for the low [GR] concentration (*, n=10), F-don signals between 600-1200 for the medium [GR] population (†**,** n=7) and F-don of >1200 for the high [GR] population (§, n=7).

**Supplementary Model S1: Mathematical model to calculate percentage monomers from FRET data.**

A derivation carried out on corrected FRET, F-don and F-acc fluorescence from the FRET assay calculates the percentage moles of GR occurring as either monomers or homodimers prior to ligand stimulation. CFP-GR and YFP-GR monomers are defined as C = CFP-GR and Y = YFP-GR, respectively. Receptor dimerization will yield three products:

The equilibrium constants of these reactions are expressed as:

**C + C = CC Y + Y = YY C + Y = CY**

**CY CC YY**

**C x Y C x C Y x Y**

**K_eq_ = = =**  **(1)**

The total molar concentration of CFP-GR (Ctot) or YFP-GR total (Ytot) in a cell expressing both receptors at the same level consists of a monomeric, heterodimeric and homodimeric fraction.

**Ctot = C + CY + CC (2)**

**Ytot = Y + CY + YY (3)**

From (2) and (3) we can derive the concentrations of the monomers and express their equations as such:

**C = Ctot - CY - CC (4)**

**Y = Ytot - CY - YY (5)**

Our FRET experiments generated fluorescence data for total CFP-GR (F-don), total YFP-GR (F-acc) as well as for corrected FRET which represents heterodimer formation. We have defined the terms as Ctot = F-don, Ytot = F-acc and CY = FRET. Radioactive binding assays reveal similar expression levels of pCFP-GR, pYFP-GR and pGRwt. As equal amounts of CFP-GR and YFP-GR plasmids were transfected we assume that the total average GR content at each receptor level is made up of one half CFP-GR and one half YFP-GR. In order to express the fluorescent signal as a molar concentration we converted fmol GR/mg protein results from saturation binding of the GRwt to nM of GR per cell at low, medium and high GR levels taking into account an experimentally derived average COS-1 cell protein content (6.49 x 10^-7^mg/cell) and volume (4.02 x 10^-9^ml/cell).

**Table S1. Average GR concentrations per cell at each GR concentration**

|  | **Average GR concentration (nM)** | | |
| --- | --- | --- | --- |
|  | **Low [GR]** | **Medium [GR]** | **High [GR]** |
| **GR/cell** | 10.8 | 24.6 | 45.8 |
| **Ctot or Ytot/cell** | 5.4 | 12.3 | 22.9 |
| **Maximal CY** | 1.8 | 4.1 | 7.6 |

Assuming 100 percent dimerization following 25 minutes of 10^-6^M DEX stimulation (Fig.4C) [1,2] one third of all receptor associations will be heterodimeric (equation 2 or 3) consisting of two differentially tagged GR monomers. This results in a maximal heterodimer concentration which is 1/6^th^ of the total monomeric GR concentration (Table S1):

**3C + 3Y CY + CC + YY**

**100%%**

**6moles 1mole 1mole 1mole**

**3moles**

We have taken the F-don (CFP-GR) and F-acc (YFP-GR) values at the initial time point (0 minutes) of the FRET experiment to represent Ctot and Ytot, respectively. These values were converted to nM concentrations for each individual cell by dividing them by the average fluorescence for each flourophore in their population and multiplying the result by the respective Ctot or Ytot/cell concentrations expressed in Table S1. The concentration of heterodimer prior to stimulation represents the uninduced (0 minute) FRET signal normalized to the averaged maximal FRET signal consisting of the time points 26 to 30 minutes after DEX induction and multiplied by the maximal molar concentration of CY heterodimer for each respective cell in relation to the average concentration expressed per population in Table S1.

Homodimer concentration cannot be measured in this system. However, using equation 1, homodimer concentration may be expressed in terms of the measurable heterodimer (corrected FRET), Ctot and Ytot concentrations. Simultaneous solution of equations (1), (4) and (5) generates C as well as Y monomer concentrations expressed as a function of Ctot (F-don), Ytot (F-acc) and corrected CY (FRET).

**3CY^2^ + (Ytot + Ctot)CY - CtotYtot**

**CY + Ytot**

**C = Y = -**

Once the concentrations of CFP-GR and YFP-GR monomers are derived it is possible to calculate the percentage monomers from the total GR concentration (Ctot + Ytot):

**C + Y**

**Ctot + Ytot**

**% GR monomers = x 100**

As the GR exists either as a monomer or a dimer we can calculate the percentage molar concentration of ligand uninduced GR dimers as such:

**% GR dimers = 100% - % GR monomers**

**Table. S2.** **GR levels and the ability to dimerize influences potency (log EC_50_) of transactivation in a range of ligands.** Cells were transfected with GRwt or GRdim (low or medium levels) and pTAT-GRE2-Elb-luc. Cells were induced with ethanol or a range (10^-12^ M to 10^-5^M) of F, MPA, or RU486 for 24h. Luciferase activity was determined and relative light units normalized against protein concentrations. Sigmoidal dose-response curves where fitted to the experimental data which generated the potency (Log EC_50_), maximal induction (Bmax) and fold-induction. Statistical analysis was carried out on logEC_50_-values using one-way ANOVA followed by Newman-Keuls post-test: (*P<0.05, **P<0.01, ***P<0.001) to compare GRwt and GRdim to the low GRwt condition and (^†^P<0.05, ^††^P<0.01, ^†††^P<0.001) to compare low GRwt against low GRdim or medium GRwt against medium GRdim. All results represent a minimum of three independent experiments performed in triplicate (±SEM).

| **Transactivation**  **parameters** | **Test**  **Compound** | **GRwt concentration** | |  | **GRdim concentration** | |
| --- | --- | --- | --- | --- | --- | --- |
|  |  | **Low** | **Medium** |  | **Low** | **Medium** |
| **Potency**  **(log EC_50_)** | **F** | -8.78 ± 0.17 | -10.48 ± 0.52* |  | -9.38 ± 0.16 | -8.94 ± 0.26^†^ |
|  | **MPA** | -7.63 ± 0.09 | -8.06 ± 0.06* |  | -7.35 ± 0.14 | -7.47 ± 0.14^†^ |
|  | **RU486** | -8.73 ± 0.24 | -9.14 ± 0.29 |  | -8.62 ± 0.10 | -8.86 ± 0.08 |
| **Maximal induction**  **(RLU/mg protein)** | **F** | 2.8 x 10^4^ ± 7.2 x 10^3^ | 8.8 x 10^4^ ± 6 x 10^3^ |  | 3.7 x 10^4^± 8.3 x 10^3^ | 2.2 x 10^5^ ± 5.7 x 10^4^ |
|  | **MPA** | 3.5 x 10^4^ ± 8.3 x 10^3^ | 1.5 x 10^5^ ± 2.3 x 10^4^ |  | 7.4 x 10^4^ ± 1.7 x 10^4^ | 6.3 x 10^5^ ± 1.3 x 10^5^ |
|  | **RU486** | 1.8 x 10^4^ ± 3.7 x 10^3^ | 6.4 x 10^4^ ± 7.5 x 10^3^ |  | 7.1 x 10^4^ ± 8.7 x 10^3^ | 4.0 x 10^5^ ± 7.8 x 10^4^ |
| **Fold-induction** | **F** | 16.02 ± 0.26 | 22.10 ± 5.43 |  | 16.02 ± 7.58 | 62.52 ± 9.90 |
|  | **MPA** | 21.43 ± 4.77 | 30.63 ± 12.21 |  | 17.12 ± 5.39 | 37.03 ± 6.25 |
|  | **RU486** | 11.82 ± 2.52 | 24.42 ± 9.23 |  | 9.267 ± 2.92 | 36.07 ± 11.72 |

**Figure S2.** **Un-induced transactivation increases and fold-induction decrease at higher GRwt concentration through single GRE.** Cells were transfected with GRwt or GRdim (low or medium levels) and 3000ng p∆ODLO, a promoter-reporter containing a single GRE. Cells were induced with ethanol, 10^-6^M DEX, F, MPA or RU486 for 24 hours. Luciferase activity was determined and relative light units (RLU) were normalized against protein concentrations. (***A***) Un-induced RLU/mg protein values following 24 hours ethanol stimulation. Statistical analysis was through two tailed unpaired t tests of low GRwt concentration against medium GRwt concentration (^†††^P<0.001), low GRdim concentration against medium GRdim concentration (^§§§^P<0.001) and GRwt against GRdim (**P<0.01, ***P<0.001). (***B***) Maximal induction and (***C***) fold-induction (calculated as maximal induction normalized to un-induced induction) were plotted. Statistical analysis was through one-way ANOVA followed by Dunnett’s post-test comparing un-induced (ethanol) conditions to the ligand-induced conditions within the low (^†††^P<0.001) or medium (^§^P<0.05, ^§§^P<0.05) concentration populations of GRwt or GRdim and two tailed unpaired t tests of ligand-induced low GRwt concentration against medium GRwt concentration (*P<0.05, **P<0.01, ***P<0.001). All results represent two experiments performed in triplicate (±SEM).

References:

1. Wrange O, Eriksson P, Perlmann T. (1989) The purified activated glucocorticoid receptor is a homodimer. The Journal of Biological Chemistry 264: 5253-5259.

2. Segard-Maurel I, Rajkowski K, Jibard N, Schweizer-Groyer G, Baulieu EE, et al. (1996) Glucocorticosteroid receptor dimerization investigated by analysis of receptor binding to glucocorticosteroid responsive elements using a monomer-dimer equilibrium model. Biochemistry 35: 1634-1642.
